# High-Deductible Health Plans and Mortality Among Cancer Survivors

**DOI:** 10.1001/jamanetworkopen.2025.56451

**Published:** 2026-01-29

**Authors:** Justin M. Barnes, Arjun Gupta, Meera Ragavan, Patricia Mae Santos, September Wallingford, Fumiko Chino

**Affiliations:** 1 Department of Radiation Oncology, Mayo Clinic, Rochester, Minnesota; 2 Costs of Care, Cambridge, Massachusetts; 3 Division of Hematology, Oncology & Transplantation, University of Minnesota, Minneapolis; 4 Division of Research, Kaiser Permanente Northern California, Pleasanton; 5 Division of Health Services, Outcomes, and Policy, Department of Radiation Oncology, Emory University, Winship Cancer Institute, Atlanta, Georgia; 6 Department of Health Policy and Management, Emory Rollins School of Public Health, Atlanta, Georgia; 7 Division of Radiation Oncology, The University of Texas MD Anderson Cancer Center, Houston; 8 Winship Cancer Institute, Atlanta, Georgia

## Abstract

**Importance:**

Patients with cancer have high utilization of health care services, and many have high financial burdens from care. High-deductible health plans (HDHPs), which offer lower premiums at the expense of higher deductibles and possibly higher out-of-pocket costs, are associated with decreased care utilization among survivors of cancer. However, it is unclear whether HDHPs are associated with cancer outcomes.

**Objective:**

To determine whether HDHP status is associated with survival among cancer survivors and individuals without history of cancer and whether financial barriers to care mediate associations between HDHP status and survival.

**Design, Setting, and Participants:**

This cross-sectional study included US adults ages 18 to 84 years who participated in the National Health Interview Survey from 2011 to 2018. Data were analyzed from December 14, 2023, to December 1, 2025.

**Exposure:**

HDHP status.

**Main Outcomes and Measures:**

Outcomes of interest were overall survival (OS) and cancer-specific survival (CSS). Analyses were adjusted for insurance status, marital status, sex, comorbidities, education, household income, geographic region, and cancer site and time since cancer diagnosis (if applicable).

**Results:**

A total of 147 254 respondents (mean [SD] age, 45.7 [16.4] years; 79 261 [50.9%] female) were identified, including 9799 (5.9%) with history of cancer. A total of 2331 cancer survivors (25.6%) and 37 473 individuals without history of cancer (28.5%) reported an HDHP at the time of NHIS participation. HDHP status was associated with worse OS (hazard ratio [HR], 1.46; 95% CI, 1.19-1.79) and CSS (HR, 1.34; 95% CI, 1.01-1.77) among cancer survivors. In contrast, among adults without history of cancer, HDHP status was not significantly associated with OS (HR, 1.08; 95% CI, 0.96-1.21; *P* for interaction = .001). In mediation analyses, HDHP status among cancer survivors was associated with increased financial barriers to care, which mediated approximately 30% to 70% of the total association of HDHP with survival.

**Conclusions and Relevance:**

In this cross-sectional study of nationwide population-based data, HDHPs were associated with worse OS and CSS among cancer survivors. However, HDHPs were not associated with mortality in adults without history of cancer. These data suggest that insurance coverage that financially discourages medical care may financially discourage necessary care and ultimately worsen cancer outcomes.

## Key Points

**Question:**

Is being insured by a high-deductible health plan associated with survival among cancer survivors?

**Findings:**

In this cross-sectional study using nationwide data from 147 254 respondents in the National Health Interview Survey, high-deductible health plan status was associated with worse overall survival and cancer-specific survival. In contrast, high deductible health plan status was not associated with survival among individuals without a cancer history.

**Meaning:**

This cross-sectional study found that high-deductible health plans were associated with worse survival for cancer survivors, perhaps because they financially disincentivized necessary medical care.

## Teaser

This cross-sectional study uses nationally representative survey data to examine the associations of high-deductible health plan status with survival among cancer survivors and individuals without a history or cancer.

## Introduction

Patients with cancer have high utilization of health care services and have known significant financial burdens from care.^1,2,3^ Unfortunately, health care costs in the US continue to increase, with increasing financial burdens on patients and families.^4^ As prices increase, insurers have shifted costs to patients via higher premiums or increased cost-sharing burdens from deductibles and coinsurance. High-deductible health plans (HDHPs)—low-premium plans that can be financially attractive for individuals who anticipate having few medical concerns—have increased in prevalence from approximately 15% in 2007 to approximately 40% in 2023 among individuals with employer-sponsored insurance.^5,6^ Additionally, 5% to 10% of Medicare enrollees use a form of HDHPs, often through supplemental high-deductible Medigap policies.^7^ However, if individuals insured by an HDHP do face a medical concern, costs are shifted to the individual until the high deductible is met, and they face potentially catastrophic financial consequences.^8^

Individuals with HDHPs have decreased or delayed health care utilization, including for evidence-based, guideline-concordant care.^9,10,11,12,13,14^ While cancer screening is not typically subject to cost sharing and thus minimally impacted by HDHP status,^15^ patients with cancer insured by HDHPs have known delays in diagnostic workup, resulting in delays in cancer treatment initiation.^16,17^ Among survivors of cancer, HDHPs have been associated not only with decreased or delayed health utilization, but also with higher out-of-pocket costs,^8,16,18^ and even small increases in patient out-of-pocket costs can lead to reduced adherence to cancer treatment.^19,20,21^

Despite documented harms of HDHPs, it remains unclear whether there is an association between HDHPs and survival among patients with cancer. Our objectives were to quantify associations between HDHP and survival among cancer survivors, compared with individuals without history of cancer, and determine whether financial barriers to care mediate survival differences.

## Methods

### Study Population

For this cross-sectional study, individuals ages 18 to 84 years with non-Medicaid insurance were identified using the 2011 to 2018 National Health Interview Survey (NHIS), an annual nationwide survey of the noninstitutionalized US population.^22^ We also obtained linked mortality files from the National Death Index (NDI). These files are the most recent available linkage of NHIS-NDI data, including mortality events through December 31, 2019. Given the deidentified nature of these publicly available data, this research was deemed exempt from oversight and informed consent by the Mayo Clinic institutional review board. Cancer survivor status was based on the question “ever told by a doctor you had cancer,” and individuals with nonmelanoma skin cancer only were excluded. This study is reported following the Strengthening the Reporting of Observational Studies in Epidemiology (STROBE) reporting guideline.

### Statistical Analysis

HDHP status was defined as an affirmative response to a survey question assessing yearly deductible at least $1200 to $1350 for an individual or at least $2400 to $2700 for a family (values depended on survey year and increased over time). These cutoffs are based on Internal Revenue Service definitions,^23^ which were integrated into the NHIS questionnaires. HDHPs are sometimes combined with a health savings account (HSA), a tax-advantaged account where HDHP enrollees can set aside money to pay for medical costs. In addition to our main analyses studying HDHP (with or without HSA), we also considered HDHPs with HSAs and HDHPs without HSAs separately. The primary end points included overall survival (OS) and cancer-specific survival (CSS; ie, freedom from death from any cancer) using age as the time scale (ie, time 0 = birth), the preferred time scale for these survey data.^24^ To more explicitly control for age in our models, we conducted sensitivity analyses using time since cancer diagnosis as the time scale (ie, time 0 = year of cancer diagnosis).

Cox proportional hazards evaluated whether HDHP status was associated with OS and CSS among cancer survivors and among individuals without history of cancer. Death from cancer as recorded in the NDI includes death from any cancer; survey respondents who had no cancer history at the time of the survey could subsequently develop a fatal cancer captured by the NDI, and survivor respondents could similarly develop a metachronous fatal malignant neoplasm. We used expanded models to assess potential interactions between HDHP status and survivor status to assess whether having a cancer history modified potential associations between HDHP status and survival. Models accounted for the NHIS survey design and survey weights. All models were adjusted for insurance status, marital status, sex, comorbidities, education, household income, geographic region, survey year, and cancer site and time since cancer diagnosis (if applicable). Comorbidity status was a composite measure based on self-reported history of diabetes, liver disease, kidney disease, hypertension, asthma, chronic obstructive pulmonary disease, heart failure, stroke, or arthritis.^25^ We conducted analyses overall and by sociodemographic subgroups, and we used expanded models with interaction terms between HDHP status and the sociodemographic factors to evaluate for heterogeneous associations of HDHPs with survival between subgroup levels. Sensitivity analyses using time since cancer diagnosis as the time scale additionally used age at diagnosis as a covariate. The plausibility of the proportional hazards assumption was assessed visually and with formal testing, and was satisfactory for all reported analyses. *P* values were 2-sided, and statistical significance was set at *P* < .05. Analyses were conducted using R software version 4.2.1 (R Project for Statistical Computing) from December 14, 2023, to December 1, 2025.

We conducted mediation analyses to determine whether financial barriers to care mediated any associations between HDHPs and survival.^26^ Financial barriers to care included delayed or forgone care due to costs in the past 12 months, cost-related medication underuse in the past 12 months,^27^ worry about paying medical bills in the case of getting sick or having an accident, and general financial worry (ie, very worried [on a Likert scale] about money for retirement, medical costs, maintaining standard of living, costs of health care, paying for children’s college, paying monthly bills, paying housing costs, and/or credit card payments).^8,27^ In our mediation analyses, our assumed causal model (eFigure 1 in Supplement 1) is that HDHPs are associated with survival, that HDHPs are associated with financial barriers,^8^ and that the financial barriers are associated with survival.^28,29^ As such, financial barriers could represent an indirect pathway by which HDHPs mediate survival. Details on how the indirect (mediating) associations and the proportion mediated (of the total HDHP effect on survival) were calculated are provided in the eMethods in Supplement 1. Each potential financial barrier was considered separately. Reporting of the mediation analyses was done in accordance with the Guideline for Reporting Mediation Analyses of Randomized Trials and Observational Studies (AGReMA) guidelines.^26^

## Results

A total of 147 254 respondents (mean [SD] age, 45.7 [16.4] years; 79 261 [50.9%] female) were identified (Table 1; eFigure 2 and eTable 1 in Supplement 1), representing 195 955 861 US adults after accounting for survey weights. Of these, 9799 individuals (5.9%) were cancer survivors, representing 11 520 647 US cancer survivors. Approximately 2331 cancer survivors (25.6%) and 37 473 individuals without history of cancer (28.5%) reported being insured by an HDHP at the time of NHIS survey. Individuals with HDHPs were more likely to be younger and have private insurance, a college degree, higher income, and no comorbidities (Table 1).

**Table 1.** Characteristics of the Study Population

Characteristic	Respondents, No. (%)			
	History of cancer		No history of cancer	
	Not HDHP (n = 7468)	HDHP (n = 2331)	Not HDHP (n = 99 982)	HDHP (n = 37 473)
Age, y				
65-84	4365 (52.5)	575 (20.7)	18 091 (13.8)	2591 (5.2)
40-64	2709 (41.7)	1542 (69.4)	43 968 (45.5)	21 000 (55.2)
18-39	394 (5.7)	214 (9.9)	37 923 (40.7)	13 882 (39.5)
Race and ethnicity				
Non-Hispanic White	6272 (85.3)	1981 (85.2)	67 603 (68.8)	28 644 (76.7)
Non-Hispanic Black	551 (6.3)	137 (5.3)	11 592 (10.7)	2814 (7.1)
Non-Hispanic other^a^	301 (4)	99 (4.3)	9037 (8.4)	2797 (7.3)
Hispanic	344 (4.4)	114 (5.2)	11 750 (12.1)	3218 (8.9)
Insurance				
Private	3214 (48.3)	1801 (80.8)	80 326 (84.5)	35 289 (95.5)
Medicare	4085 (49.4)	530 (19.2)	16 411 (12.5)	2184 (4.5)
Other/not reported	169 (2.3)	0 (0)	3245 (3)	0 (0)
Marital status				
Not married	4032 (67.5)	1308 (68)	50 068 (58.5)	20 855 (64.1)
Married	3428 (32.4)	1018 (31.9)	49 745 (41.4)	16 567 (35.8)
Not reported	8 (0.1)	5 (0.2)	169 (0.1)	51 (0.1)
Sex				
Male	2897 (41.4)	852 (38.6)	46 389 (49.5)	17 855 (50)
Female	4571 (58.6)	1479 (61.4)	53 593 (50.5)	19 618 (50)
Region				
Northeast	1474 (21.7)	349 (15.2)	17 971 (20.3)	5339 (14.8)
Midwest	1909 (25)	646 (28.2)	22 717 (22.9)	10 421 (28.1)
South	2408 (33.6)	768 (36.3)	33 944 (34)	12 736 (36.5)
West	1677 (19.8)	568 (20.3)	25 350 (22.8)	8977 (20.7)
Education				
College degree	3490 (48.4)	1265 (55.2)	50 291 (50.2)	21 786 (57.8)
Completed high school	3334 (43.7)	961 (40.9)	42 660 (42.9)	14 014 (37.5)
No high school degree	644 (7.9)	105 (3.9)	7031 (6.9)	1673 (4.7)
Poverty				
≥400% FPL	3265 (49.2)	1227 (56.8)	44 095 (48.4)	19 710 (55.9)
250%-399% FPL	1668 (21.1)	549 (23.1)	21 983 (21.6)	8662 (22.2)
125%-249% FPL	1360 (15.1)	343 (11.8)	17 236 (15.4)	5339 (12.7)
<125% FPL	393 (3.9)	83 (2.6)	8998 (6.7)	1669 (3.4)
Unknown/not reported	782 (10.7)	129 (5.7)	7670 (7.9)	2093 (5.8)
Comorbidities				
No	1770 (25.1)	796 (36.1)	57 143 (60.1)	22 397 (61.9)
Yes	5698 (74.9)	1535 (63.9)	42 839 (39.9)	15 076 (38.1)
Year				
2011-2013	2745 (36.6)	723 (29)	40 540 (38)	11 993 (29.8)
2014-2016	3020 (37.4)	986 (38.7)	40 351 (38)	15 779 (38.9)
2017-2018	1703 (26)	622 (32.4)	19 091 (23.9)	9701 (31.4)
Cancer type				
Breast	1638 (21)	485 (20.1)	NA	NA
Cervix	404 (5.3)	185 (7.7)	NA	NA
Colon	383 (5.1)	83 (4)	NA	NA
Lung	155 (2)	34 (1.3)	NA	NA
Melanoma	614 (8.5)	225 (9.8)	NA	NA
Multiple	1197 (15.9)	326 (13.3)	NA	NA
Other/not reported	2154 (29.6)	786 (35)	NA	NA
Prostate	923 (12.7)	207 (8.8)	NA	NA
Time since cancer diagnosis				
≥16 y	1694 (21.9)	474 (18.9)	NA	NA
7-15 y	2249 (29.9)	664 (28.2)	NA	NA
3-6 y	1718 (23.8)	562 (24.5)	NA	NA
0-2 y	1706 (22.9)	615 (27.7)	NA	NA
Unknown/not reported	101 (1.5)	16 (0.7)	NA	NA

Abbreviations: FPL, federal poverty level; HDHP, high-deductible health plan.

^a^ Other includes American Indian or Alaska Native only, Asian only, multiple races, and all other race groups.

**Figure 1.**
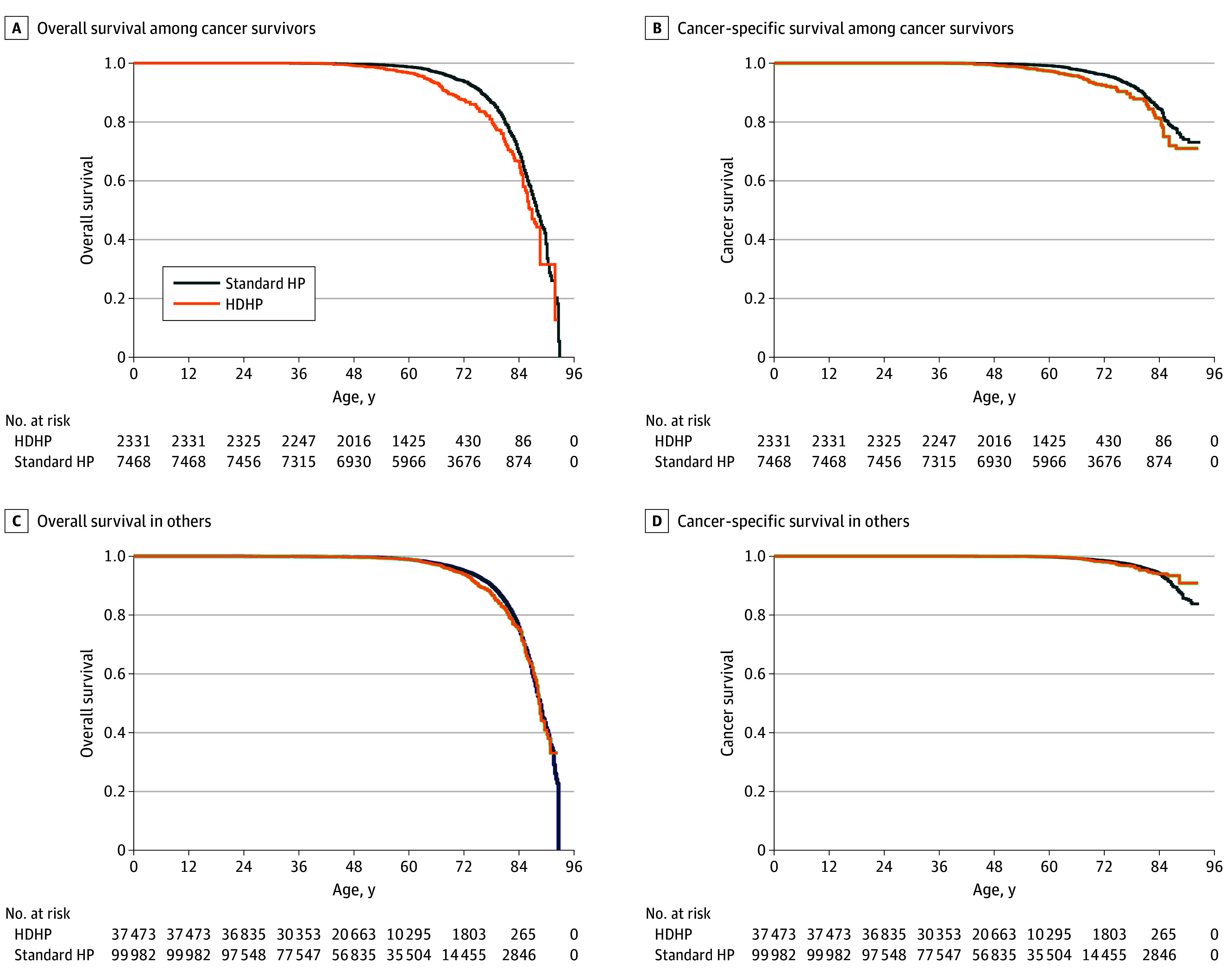
Overall and Cancer-Specific Survival for Patients With and Without History of Cancer The time scale is age at the time of death or age at the end of study follow-up (2019). Cancer-specific survival can include cancer death from a cancer that was diagnosed after the time of survey completion. HDHP indicates high-deductible health plan.

**Figure 2.**
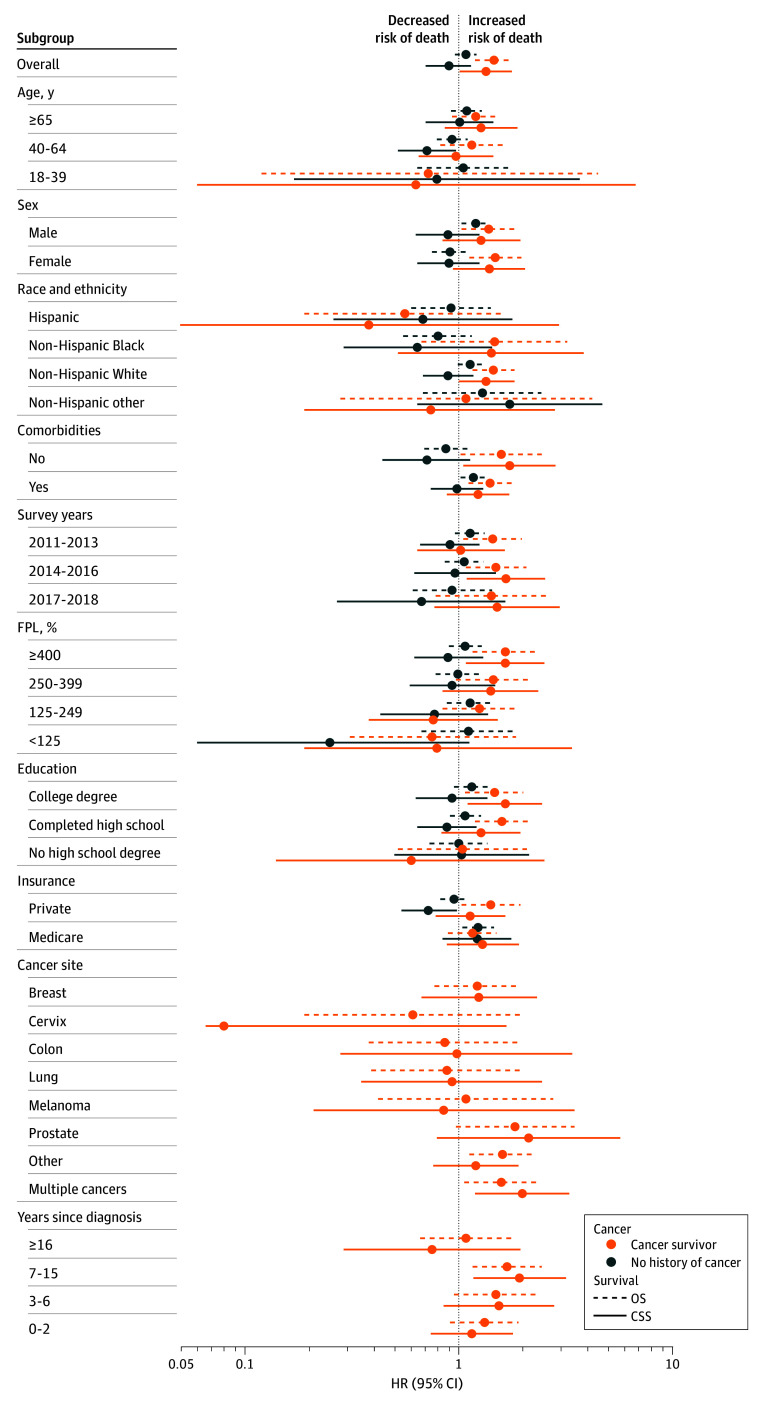
Associations of High Deductible Health Plans (HDHPs) With Overall Survival (OS) and Cancer-Specific Survival (CSS) Overall and by Subgroups Cancer-specific survival can include cancer death from a cancer that was diagnosed after the time of survey completion. Corresponding hazard ratios (HRs), 95% CIs, and *P* values are provided in eTables 2-4 in Supplement 1. Other race and ethnicity includes American Indian or Alaska Native only, Asian only, multiple races, and all other race groups. FPL indicates federal poverty level.

HDHP status was associated with worse OS (hazard ratio [HR], 1.46; 95% CI, 1.19-1.79) and CSS (HR, 1.34; 95% CI, 1.01-1.77) among cancer survivors overall in adjusted models (Figure 1 and Figure 2; eTable 2 and eTable 3 in Supplement 1). We found no evidence of significant heterogeneity in the associations between OS and HDHP status between subgroups. Notably, however, HDHP status was associated with worse OS when limiting the sample to individuals with private insurance (HR, 1.41; 95% CI, 1.03-1.94). HDHP status was associated with worse CSS among cancer survivors who had income at least 400% of the federal poverty level (HR, 1.65; 95% CI, 1.08-2.51; *P* for interaction = .03) and had college education (HR, 1.65; 95% CI, 1.10-2.45; *P* for interaction = .04) (Figure 2; eTable 2 and eTable 3 in Supplement 1). There were less clear patterns by cancer site (Figure 2; eTable 4 in Supplement 1). In sensitivity analyses using time since diagnosis as the time scale and enabling adjustment for age as a covariate, we similarly found worse OS for cancer survivors with HDHPs (HR, 1.21; 95% CI, 1.00-1.47), with less evidence for an association of HDHP status with CSS (HR, 1.20; 95% CI, 0.92-1.55).

In contrast, among adults without a history of cancer at the time of the NHIS survey, HDHP status was not significantly associated with OS (HR, 1.08; 95% CI, 0.96-1.21) or CSS (HR, 0.90; 95% CI, 0.70-1.14). The association of HDHPs and OS (*P* for interaction = .001) and CSS (*P* for interaction < .001) was significantly worse for cancer survivors relative to individuals without a cancer history (Figure 2; eTable 2 and eTable 3 in Supplement 1).

In assessing our hypothesized causal model for mediation analyses, HDHP status among cancer survivors was associated with increased general financial worry (odds ratio [OR], 1.58; 95% CI, 1.33-1.89), increased delayed or forgone care due to cost (OR, 2.08; 95% CI, 1.67-2.58), increased cost-related medication underuse (OR, 1.92; 95% CI, 1.53-2.40), and increased worry about medical bills (OR, 1.79; 95% CI, 1.45-2.22), and these factors were each associated with worse OS and CSS (Table 2). General financial worry, delayed or forgone care due to cost, cost-related medication underuse, and worry about medical bills were statistically significant mediators of the associations of HDHP with OS and CSS, each mediating approximately 30% to 70% of the total association (Table 2).

**Table 2.** Mediation Analyses Evaluating the Role of Barriers to Care on the Associations Between HDHP and Survival^a^

Barrier to care	Association between (95% CI)				Mediation analysis		
	HDHP and barrier to care, OR	HR			HR (95% CI)	*P* value	% Mediated
		Barrier to care and survival	HDHP and survival				
			Adjusted for barrier to care	Unadjusted for barrier to care			
**Overall survival**							
General financial worry	1.58 (1.33-1.89)	1.65 (1.27-2.16)	1.51 (1.17-1.94)	1.46 (1.19-1.79)	1.26 (1.08-1.46)	.003	36
Delayed or forgone care	2.08 (1.67-2.58)	2.15 (1.55-2.99)	1.41 (1.14-1.74)	1.46 (1.19-1.79)	1.75 (1.31-2.34)	<.001	62
Cost-related medication underuse	1.92 (1.53-2.4)	1.94 (1.5-2.52)	1.43 (1.16-1.76)	1.46 (1.19-1.79)	1.54 (1.23-1.93)	<.001	55
Worry about medical bills	1.79 (1.45-2.22)	1.96 (1.5-2.56)	1.45 (1.18-1.78)	1.46 (1.19-1.79)	1.48 (1.2-1.83)	<.001	51.4
**Cancer-specific survival**							
General financial worry	1.58 (1.33-1.89)	1.52 (1.07-2.18)	1.55 (1.12-2.15)	1.34 (1.01-1.77)	1.21 (1.01-1.45)	.035	30.6
Delayed or forgone care	2.08 (1.67-2.58)	2.24 (1.49-3.37)	1.29 (0.98-1.71)	1.34 (1.01-1.77)	1.8 (1.28-2.55)	.001	69.8
Cost-related medication underuse	1.92 (1.53-2.4)	1.99 (1.44-2.75)	1.3 (0.98-1.73)	1.34 (1.01-1.77)	1.57 (1.21-2.03)	.001	62.9
Worry about medical bills	1.79 (1.45-2.22)	2.32 (1.62-3.32)	1.32 (1-1.74)	1.34 (1.01-1.77)	1.63 (1.24-2.15)	<.001	63.8

Abbreviation: HR, hazard ratio.

^a^ All analyses were adjusted for covariates, including insurance status, marital status, sex, comorbidities, education, household income, geographic region, survey year, cancer site, and time since cancer diagnosis.

In analyses considering HSA status, 7.6% of cancer survivors had an HDHP with an HSA and 18.0% had an HDHP without an HSA (eTable 5 in Supplement 1). Cancer survivors with HDHP plans with HSAs had worse survival compared with those with other plans (OS: HR, 1.68; 95% CI, 1.03-2.74; CSS: HR, 1.42; 95% CI, 0.84-2.41), and cancer survivors with HDHP plans without HSAs also had worse survival compared with those with other plans (OS: HR, 1.36; 95% CI, 1.10-1.69; CSS: HR, 1.27; 95% CI, 0.93-1.72) (eTables 6-8 in Supplement 1). In mediation analysis examining HDHPs without HSAs, results were very similar to the overall mediation analyses (eTable 9 in Supplement 1). However, HDHP with HSA status was not associated with general financial worry, delayed or forgone care due to cost, or cost-related medication underuse, nor were these factors statistically significant mediators of the association of HDHP with HSA status with survival. However, HDHP with HSA status was associated with increased worry about medical bills (OR, 1.46; 95% CI, 1.04-2.04), which mediated 27.6% and 41.2% of the association of HDHP with HSA with OS (*P* = .04) and CSS (*P* = .049), respectively (eTable 9 in Supplement 1).

## Discussion

In this nationwide cross-sectional study of population-based survey data, our findings suggest that self-reported HDHP status was associated with worse OS and CSS among cancer survivors, regardless of HSA status. Financial barriers to care, such as delaying or forgoing care due to cost, underusing medications due to cost, being worried about potential future medical bills, and general financial concerns, were significant mediators of the association between HDHPs and survival among cancer survivors. This danger appears to be unique to cancer survivors, as HDHPs were not associated with survival among adults without a cancer history.

Prior research has demonstrated that women with breast cancer enrolled in HDHPs are more likely to experience delays in imaging, biopsy, and diagnosis, which have downstream implications on timely initiation of treatment.^16^ Prior work also has shown that patients with cancer and HDHPs have nearly 70% higher out-of-pocket medical expenditures compared with individuals with standard plans.^18^ Finally, cancer survivors with HDHPs have been reported to be more likely to forgo or delay care due to costs.^8^ Our findings add to the literature by demonstrating the negative associations for cancer survivors for downstream outcomes like survival, which seems to be in part mediated by the financial strain associated with HDHPs.

Financial strain that could be caused by increased out-of-pocket expenditures experienced by patients with cancer could be associated with survival in at least 2 ways. First, financial pressures can lead to changes in health-seeking behavior. For example, even small changes in out-of-pocket costs are associated with changes in adherence to cancer treatment^19,20^ which could compromise efficacy and chance of cure. Beyond cancer treatment itself, impaired health-seeking behavior at any part of the survivorship continuum could lead to worse outcomes. Examples include delaying in initial diagnosis due to hesitation to seek care for initial cancer workup, delaying or avoiding cancer treatment, missing follow-up visits for surveillance imaging for early detection and management of potential cancer recurrence, or delaying or forgoing other nononcologic medical care for other conditions, which may have been caused or exacerbated by cancer treatment.^3^ Prior research shows that other insurance policies that may impair access to care or increase financial pressures, such as short-term plans and policies that disrupt the health insurance market, have been associated with fewer early-stage cancer diagnoses and higher rates of delayed and forgone care among patients with cancer.^27,30,31^ In contrast, health care policies that bolster access to care and decrease costs, such as Medicaid expansion, have been associated with improved cancer outcomes, some of which seem to be driven by earlier cancer detection.^32,33,34,35,36^ This potential mechanism is also supported by our mediation analyses, in which delayed or forgone care due to cost and cost-related medication underuse were significant mediators of the association of HDHPs with survival. Additionally, given that higher health care utilization is expected in cancer survivors relative to the general US population, hence greater potential for inadequate health care utilization among cancer survivors, this potential mechanism could also explain why HDHPs were associated with survival among cancer survivors but were not associated with survival in adults without a cancer history.^37,38,39^

Second, HDHP status may lead to a greater degree of patient-reported financial toxic effects. Our data demonstrated that HDHPs were associated with increased risk of financial worry and cost-driven changes in behavior, and that these finances-related issues were significant mediators of the association between HDHPs and survival. That financial worry driven by potentially increased costs for care under an HDHP could ultimately lead to changes in survival is consistent with prior data, which suggest that severe financial hardship is associated with an increased risk of mortality among patients with cancer.^28^ While the mechanisms by which financial hardship is associated with mortality are not clear, decreased health-seeking behaviors could contribute, and an evolving body of research additionally suggests that socioeconomic stressors may lead to altered molecular pathways and gene expression, which are associated with more aggressive tumor biology and higher risk of mortality.^40,41^

In contrast to prior work suggesting that HSAs could mitigate some of the negative associations of HDHP with health care utilization,^8^ our data showed similar adverse associations of HDHPs for individuals with and without HSAs. The reasons for this discrepancy are unclear. One possibility is the small amount of mitigation of financial barriers to health care utilization with HSA in the prior study was insufficient to lead to differences in survival. Interestingly, the mediators were different for individuals with and without HSAs. While all cost barriers to medical care and financial worry measures appeared to be important mediators of the associations of HDHPs with survival for individuals without HSAs, only 1 measure—worrying about medical bills if the respondent were to get sick or have an accident in the future—appeared to be an important mediator of the association of HDHPs with survival for those with HSAs. While the worrying about medical bills variable could capture underlying comorbidities predisposing respondents to increased care utilization or poor outcomes, if we assume this measure is an indicator of financial toxic effects, it could suggest that the financial toxic effects component of HDHPs is predominant rather than changes in health-seeking behaviors. This is perhaps consistent with recent reports suggesting that more than 35% of people in the US could not afford a $400 unexpected expense.^42^ Alternatively, especially in light of limited importance of the general financial worry measure, which also includes, among many other factors, concerns about paying medical bills, the importance of this factor could be driven by confounding if individuals with the higher deductibles, higher potential out-of-pocket costs, and/or lower capacity to contribute to an account are more likely to have an HSA. Notably, demographics differ between individuals with and without HSAs, likely due to a combination of insured individuals’ preferences and differences in employers that offer HSAs (typically larger firms).^43^

Cancer survivor subgroups that experienced disproportionately worse survival outcomes associated with HDHPs included individuals with higher income and higher educational attainment, which was unexpected. While it is possible that HDHPs may counteract what otherwise might have been good access to care for individuals with socioeconomic advantage, additional barriers to care would be expected to disproportionately affect those already facing socioeconomic disadvantage. Alternatively, HDHP status (which is self-reported) could be a surrogate for financial toxic effects or poor health literacy, which can still occur in individuals with higher income and education and perhaps may lead them to pursue an HDHP to minimize monthly premiums. It could also be a surrogate for baseline willingness to seek care. Notably, however, we conducted a number of subgroup analyses, and our reported CIs and *P* values were not adjusted for multiple comparisons, so these could also be spurious findings.

### Limitations

Our study is subject to a number of important limitations. First, HDHP status is self-reported and is likely inaccurate for some individuals. More than half of consumers are unsure about their annual deductible amount, and there is likely a higher prevalence of HDHP plans and a lower prevalence of HSA plans than what was self-reported in these data.^43,44^ Similarly, patients may report having private insurance even if they have Medicaid if they are part of a managed care plan. It is possible that individuals with higher medical expenses, a greater degree of financial toxic effects, or lower socioeconomic status, which are linked with adverse cancer outcomes or which may occur more frequently with more advanced cancers, are more likely to report having an HDHP. Similarly, we do not have specific plan details (including exact deductible) or out-of-pocket costs faced by the individual. Next, HDHP status is based on a single time point—the time of NHIS participation—and we do not have information about the type of insurance plans (if any) individuals had prior to the HDHP and/or at the time of their cancer diagnosis and treatment or after survey participation. Furthermore, the financial worry and cost-driven barriers to care measures are based on the time of survey, such that we are unable to capture resolving or worsening financial issues around the time of cancer diagnosis or potential mortality event. While we sought to assess potential mechanisms by which HDHP status is associated with mortality, these mechanisms remain unclear. Additionally, we lack information about cancer stage, cancer-directed therapies, recurrences, or short- or long-term complications. Similarly, cancer mortality is from any cancer and could be from a cancer that was diagnosed after NHIS participation. Furthermore, while this cohort is nationally representative of cancer survivors, certain patients with cancer, such as those with aggressive disease with limited life expectancy, are likely underrepresented in these data.

## Conclusions

In this cross-sectional study of nationwide population-based survey data, self-reported HDHP status was associated with increased risk of overall and cancer mortality among cancer survivors, regardless of HSA status. The association between HDHPs and survival was greater in cancer survivors compared with the general population, among whom HDHPs had minimal associations with survival. Contributing factors to the association of HDHPs with survival likely included financial toxic effects and delaying or forgoing necessary medical care due to costs. In the current political environment, in which there may be proliferation of HDHPs, these results suggest that HDHP proliferation could exacerbate adverse cancer outcomes. These data underscore the importance of educating patients with cancer about potential (increased) need for health care utilization, educating the public about potential risks of HDHPs, identifying policy solutions to decrease costs of care, and optimizing health policies to avoid disincentives for seeking what may turn out to be necessary and lifesaving or life-prolonging care.

## Notes

Supplement 1.
eMethods.
**eTable 1.** Comparison of included cases and excluded cases due to missing covariate information **eTable 2.** Associations of high-deductible health plans with overall survival, overall and by sociodemographic subgroups **eTable 3.** Associations of high-deductible health plans with cancer-specific survival, overall and by sociodemographic subgroups **eTable 4.** Associations of high-deductible health plans with overall and cancer survival by cancer site and time since diagnosis subgroups **eTable 5.** Characteristics of the study population with consideration of health savings account status **eTable 6.** Associations of high-deductible health plans and HSA status with overall survival, overall and by sociodemographic subgroups **eTable 7.** Associations of high-deductible health plans and HSA status with cancer-specific survival, overall and by sociodemographic subgroups **eTable 8.** Associations of high-deductible health plans and HSA status with survival by cancer site and time since diagnosis subgroups. **eTable 9.** Mediation analyses evaluating the role of barriers to care on the associations between HDHP with or without HSA and survival **eFigure 1.** Assumed causal pathway for mediation analyses **eFigure 2.** Derivation of the dataset for analyses

Supplement 2.
Data Sharing Statement
