# Supplementary material for: High-Deductible Health Plans and Mortality Among Cancer Survivors
Source: JAMA Netw Open. 2026 Jan 29;9(1):e2556451. doi: 10.1001/jamanetworkopen.2025.56451 (PMC12856683; doi:10.1001/jamanetworkopen.2025.56451)
Supplement: Supplement 1. — eMethods. eTable 1. Comparison of included cases and excluded cases due to missing covariate information eTable 2. Associations of high-deductible health plans with overall survival, overall and by sociodemographic subgroups eTable 3. Associations of high-deductible health plans with cancer-specific survival, overall and by sociodemographic subgroups eTable 4. Associations of high-deductible health plans with overall and cancer survival by cancer site and time since diagnosis subgroups eTable 5. Characteristics of the study population with consideration of health savings account status eTable 6. Associations of high-deductible health plans and HSA status with overall survival, overall and by sociodemographic subgroups eTable 7. Associations of high-deductible health plans and HSA status with cancer-specific survival, overall and by sociodemographic subgroups eTable 8. Associations of high-deductible health plans and HSA status with survival by cancer site and time since diagnosis subgroups. eTable 9. Mediation analyses evaluating the role of barriers to care on the associations between HDHP with or without HSA and survival eFigure 1. Assumed causal pathway for mediation analyses eFigure 2. Derivation of the dataset for analyses [file jamanetwopen-e2556451-s001.pdf]

Supplemental Online Content

Barnes JM, Gupta A, Ragavan M, Santos PM, Wallingford S, Chino F. High-deductible health plans and mortality among cancer survivors. *JAMA Netw Open*. 2026;9(1):e2556451. doi:10.1001/jamanetworkopen.2025.56451

eMethods.
eTable 1. Comparison of included cases and excluded cases due to missing covariate information
eTable 2. Associations of high-deductible health plans with overall survival, overall and by sociodemographic subgroups
eTable 3. Associations of high-deductible health plans with cancer-specific survival, overall and by sociodemographic subgroups
eTable 4. Associations of high-deductible health plans with overall and cancer survival by cancer site and time since diagnosis subgroups
eTable 5. Characteristics of the study population with consideration of health savings account status
eTable 6. Associations of high-deductible health plans and HSA status with overall survival, overall and by sociodemographic subgroups
eTable 7. Associations of high-deductible health plans and HSA status with cancer-specific survival, overall and by sociodemographic subgroups
eTable 8. Associations of high-deductible health plans and HSA status with survival by cancer site and time since diagnosis subgroups.
eTable 9. Mediation analyses evaluating the role of barriers to care on the associations between HDHP with or without HSA and survival
eFigure 1. Assumed causal pathway for mediation analyses
eFigure 2. Derivation of the dataset for analyses

This supplemental material has been provided by the authors to give readers additional information about their work.

© 2026 Barnes JM et al. *JAMA Network Open*.

Supplementary Methods

Mediation analyses calculations

For the mediation analyses, the proportional hazards models included a financial barrier to care covariate, the association between HDHP and the financial barrier to care were examined using logistic regression (adjusting for the same set of covariates, and also accounting for the NHIS survey design and survey weights). The indirect (mediating) effect is defined as the product of the coefficient for the barrier to care variable (in the models where the outcome was survival) and the coefficient for the high deductible health plan variable (in the models where the outcome was a barrier to care). The variance of the indirect effect, which was used to generate confidence intervals and p-values, was calculated based on the Sobel method.[1–3] We also present an estimate of the proportion mediated,[4] which is defined as proportion mediated = as (indirect effect)/(indirect effect + effect after adjusting for the barrier to care).

References

1. Sobel ME. Asymptotic Confidence Intervals for Indirect Effects in Structural Equation Models. *Sociol Methodol*. 1982;13:290. doi:10.2307/270723
2. MacKinnon DP. *Introduction to Statistical Mediation Analysis*. Taylor & Francis; 2008.
3. MacKinnon DP, Fairchild AJ, Fritz MS. Mediation analysis. *Annu Rev Psychol*. 2007;58:593-614. doi:10.1146/annurev.psych.58.110405.085542
4. Rijnhart JJM, Twisk JWR, Eekhout I, Heymans MW. Comparison of logistic-regression based methods for simple mediation analysis with a dichotomous outcome variable. *BMC Med Res Methodol*. 2019;19(1):19. doi:10.1186/s12874-018-0654-z

© 2026 Barnes JM et al. *JAMA Network Open*.

eTable 1: Comparison of included cases and excluded cases due to missing covariate information

		History of cancer		No history of cancer		History of cancer		No history of cancer	
		Not HDHP	HDHP	Not HDHP	HDHP	Not HDHP	HDHP	Not HDHP	HDHP
Age (years)	65-84	52.5	20.7	13.8	5.2	46.4	21.6	11.1	3.8
	40-64	41.7	69.4	45.5	55.2	49	75.3	47.4	54.7
	18-39	5.7	9.9	40.7	39.5	4.6	3.1	41.5	41.5
Race and ethnicity	Non-Hispanic White	85.3	85.2	68.8	76.7	83.5	79.8	64.3	73.6
	Non-Hispanic Black	6.3	5.3	10.7	7.1	4.6	9.5	9.6	5.9
	Non-Hispanic Other	4	4.3	8.4	7.3	8.6	6	11.9	11.7
	Hispanic	4.4	5.2	12.1	8.9	3.4	4.7	14.2	8.9
Insurance	Private	48.3	80.8	84.5	95.5	59.6	83.3	88.1	97.5
	Medicare	49.4	19.2	12.5	4.5	38.3	16.7	9.4	2.5
	Other/Not reported	2.3	0	3	0	2.2	0	2.5	0
Marital status	Not married	67.5	68	58.5	64.1	67	72.7	60.1	63.1
	Married	32.4	31.9	41.4	35.8	32.7	26.1	38.6	35.8
	Not reported	0.1	0.2	0.1	0.1	0.3	1.2	1.3	1.1
Sex	Male	41.4	38.6	49.5	50	31.8	18.8	47.1	43.6
	Female	58.6	61.4	50.5	50	68.2	81.2	52.9	56.4
Region	Northeast	21.7	15.2	20.3	14.8	17.6	13.2	18.8	14.7
	Midwest	25	28.2	22.9	28.1	27.4	32.2	22.3	28.4
	South	33.6	36.3	34	36.5	27.1	31.9	27.4	29.9
	West	19.8	20.3	22.8	20.7	27.9	22.7	31.5	27
Education	College degree	48.4	55.2	50.2	57.8	51.8	46	54.3	62.7
	Completed high school	43.7	40.9	42.9	37.5	41.8	54	39.3	34
	No high school degree	7.9	3.9	6.9	4.7	6.5	0	6.4	3.3
Poverty	>=400% FPL	49.2	56.8	48.4	55.9	35.5	56.3	40.8	42.7
	250-399% FPL	21.1	23.1	21.6	22.2	18.9	16	17	18.9
	125-249% FPL	15.1	11.8	15.4	12.7	11.9	6.9	11	11.8
	<125% FPL	3.9	2.6	6.7	3.4	5.8	0	3.8	1.8
	Unknown/not reported	10.7	5.7	7.9	5.8	27.9	20.8	27.4	24.8
Comorbidities	No	25.1	36.1	60.1	61.9	27.4	39.1	69.5	67.8
	Yes	74.9	63.9	39.9	38.1	72.6	60.9	30.5	32.2
Year	2011-2013	36.6	29	38	29.8	32.9	28.4	41.8	32.1

© 2026 Barnes JM et al. *JAMA Network Open*.

	2014-2016	37.4	38.7	38	38.9	35.2	23.2	34.7	39.1
	2017-2018	26	32.4	23.9	31.4	31.9	48.4	23.5	28.7
Cancer type	Breast	21	20.1	NA		28.3	47	NA	
	Cervix	5.3	7.7			4.4	0		
	Colon	5.1	4			7.5	1		
	Lung	2	1.3			6.8	3.1		
	Melanoma	8.5	9.8			7.4	1.6		
	Multiple	15.9	13.3			12.8	17.6		
	Other/Not reported	29.6	35			23.8	28.7		
	Prostate	12.7	8.8			9.2	1		
Time since cancer diagnosis	>=16 years	21.9	18.9			18.2	6.2		
	7-15 years	29.9	28.2			30.7	44.7		
	3-6 years	23.8	24.5			27.1	31.9		
	0-2 years	22.9	27.7			16.8	13.4		
	Unknown/not reported	1.5	0.7			7.2	3.9		

HDHP = high-deductible health plan, FPL = federal poverty level.

© 2026 Barnes JM et al. *JAMA Network Open*.

eTable 2: Associations of high-deductible health plans with overall survival, overall and by sociodemographic subgroups

	Cancer survivors		No history of cancer		Cancer survivors relative to those without cancer history	
Subgroup	HR (95% CI)	P-value	HR (95% CI)	P-value	HR (95% CI)	P-value
Overall	1.46 (1.19 - 1.79)	0	1.08 (0.96 - 1.21)	0.203	1.5 (1.19 - 1.89)	0.001
>=65 years	1.2 (0.93 - 1.55)	0.152	1.09 (0.92 - 1.28)	0.329	1.08 (0.8 - 1.46)	0.6
40-64 years	1.15 (0.82 - 1.61)	0.432	0.93 (0.79 - 1.1)	0.409	1.42 (0.98 - 2.07)	0.064
18-39 years	0.72 (0.12 - 4.47)	0.727	1.05 (0.64 - 1.7)	0.856	0.79 (0.16 - 3.94)	0.77
Male	1.38 (1.03 - 1.85)	0.032	1.2 (1.03 - 1.39)	0.02	1.27 (0.93 - 1.74)	0.131
Female	1.48 (1.12 - 1.96)	0.006	0.91 (0.75 - 1.09)	0.306	2.03 (1.45 - 2.85)	<.001
Non-Hispanic White	1.45 (1.16 - 1.82)	0.001	1.13 (0.99 - 1.28)	0.06	1.49 (1.16 - 1.91)	0.002
Non-Hispanic Black	1.47 (0.67 - 3.21)	0.336	0.8 (0.55 - 1.15)	0.222	1.24 (0.41 - 3.74)	0.697
Non-Hispanic Other	1.08 (0.28 - 4.2)	0.906	1.29 (0.68 - 2.43)	0.439	0.74 (0.24 - 2.25)	0.597
Hispanic	0.56 (0.19 - 1.62)	0.287	0.92 (0.6 - 1.41)	0.7	1.63 (0.47 - 5.73)	0.443
No comorbidities	1.58 (1.02 - 2.44)	0.038	0.87 (0.69 - 1.11)	0.261	2.17 (1.32 - 3.56)	0.002
Comorbidities	1.4 (1.11 - 1.77)	0.005	1.17 (1.02 - 1.34)	0.023	1.28 (0.99 - 1.66)	0.062
2011-2013 survey	1.44 (1.05 - 1.97)	0.022	1.13 (0.96 - 1.32)	0.135	1.41 (1.03 - 1.94)	0.031
2014-2016 survey	1.49 (1.08 - 2.07)	0.015	1.06 (0.86 - 1.3)	0.606	1.55 (1.05 - 2.28)	0.027
2017-2018 survey	1.42 (0.78 - 2.57)	0.254	0.93 (0.61 - 1.43)	0.744	1.53 (0.69 - 3.39)	0.3
>=400% FPL	1.65 (1.16 - 2.36)	0.006	1.07 (0.9 - 1.28)	0.434	1.95 (1.34 - 2.82)	<.001
250-399% FPL	1.45 (0.97 - 2.15)	0.068	0.99 (0.78 - 1.26)	0.953	1.44 (0.89 - 2.32)	0.142
125-249% FPL	1.25 (0.84 - 1.84)	0.268	1.13 (0.88 - 1.45)	0.327	1.03 (0.64 - 1.66)	0.896
<125% FPL	0.75 (0.31 - 1.85)	0.538	1.11 (0.67 - 1.82)	0.683	0.46 (0.1 - 2.23)	0.337
College degree	1.47 (1.07 - 2)	0.016	1.15 (0.95 - 1.38)	0.144	1.51 (1.07 - 2.12)	0.019
Completed high school	1.59 (1.19 - 2.12)	0.002	1.07 (0.91 - 1.27)	0.398	1.59 (1.13 - 2.22)	0.007
No high school degree	1.04 (0.52 - 2.08)	0.911	1 (0.73 - 1.36)	0.997	0.9 (0.41 - 1.96)	0.789
Private insurance	1.41 (1.03 - 1.94)	0.033	0.95 (0.82 - 1.11)	0.528	1.88 (1.34 - 2.64)	<.001
Medicare	1.16 (0.89 - 1.5)	0.264	1.23 (1.04 - 1.46)	0.017	0.92 (0.68 - 1.24)	0.585

HR=hazard ratio, CI = confidence interval, FPL = federal poverty level.

© 2026 Barnes JM et al. *JAMA Network Open*.

eTable 3: Associations of high-deductible health plans with cancer-specific survival, overall and by sociodemographic subgroups

	Cancer survivors		No history of cancer		Cancer survivors relative to those without cancer history	
Subgroup	HR (95% CI)	P-value	HR (95% CI)	P-value	HR (95% CI)	P-value
Overall	1.34 (1.01 - 1.77)	0.04	0.9 (0.7 - 1.14)	0.38	1.93 (1.36 - 2.76)	<.001
>=65 years	1.27 (0.86 - 1.88)	0.226	1.01 (0.7 - 1.45)	0.964	1.2 (0.74 - 1.95)	0.462
40-64 years	0.97 (0.65 - 1.45)	0.887	0.71 (0.52 - 0.97)	0.03	1.72 (1.03 - 2.87)	0.037
18-39 years	0.63 (0.06 - 6.7)	0.703	0.79 (0.17 - 3.67)	0.759	0.88 (0.06 - 13.34)	0.926
Male	1.27 (0.84 - 1.94)	0.26	0.89 (0.63 - 1.25)	0.496	1.86 (1.14 - 3.03)	0.013
Female	1.39 (0.94 - 2.04)	0.097	0.9 (0.64 - 1.25)	0.516	2.05 (1.22 - 3.44)	0.007
Non-Hispanic White	1.34 (1 - 1.82)	0.053	0.89 (0.68 - 1.17)	0.399	2.03 (1.38 - 2.99)	<.001
Non-Hispanic Black	1.42 (0.52 - 3.83)	0.493	0.64 (0.29 - 1.43)	0.279	1.42 (0.28 - 7.08)	0.672
Non-Hispanic Other	0.74 (0.19 - 2.81)	0.657	1.73 (0.64 - 4.68)	0.278	0.9 (0.16 - 5.13)	0.901
Hispanic	0.38 (0.05 - 2.94)	0.354	0.68 (0.26 - 1.78)	0.436	1.99 (0.3 - 13.06)	0.474
No comorbidities	1.73 (1.05 - 2.83)	0.031	0.71 (0.44 - 1.13)	0.149	2.83 (1.34 - 5.95)	0.006
Comorbidities	1.23 (0.88 - 1.72)	0.23	0.98 (0.74 - 1.3)	0.908	1.59 (1.07 - 2.36)	0.021
2011-2013 survey	1.02 (0.64 - 1.64)	0.934	0.91 (0.66 - 1.25)	0.557	1.43 (0.88 - 2.32)	0.151
2014-2016 survey	1.66 (1.09 - 2.53)	0.017	0.96 (0.62 - 1.49)	0.871	2.18 (1.17 - 4.07)	0.014
2017-2018 survey	1.51 (0.77 - 2.96)	0.234	0.67 (0.27 - 1.65)	0.387	2.7 (0.88 - 8.3)	0.083
>=400% FPL	1.65 (1.08 - 2.51)	0.02	0.89 (0.62 - 1.3)	0.551	2.63 (1.59 - 4.35)	<.001
250-399% FPL	1.41 (0.84 - 2.35)	0.193	0.93 (0.59 - 1.48)	0.757	1.79 (0.87 - 3.68)	0.113
125-249% FPL	0.76 (0.38 - 1.52)	0.446	0.77 (0.43 - 1.37)	0.37	1.33 (0.57 - 3.06)	0.51
<125% FPL	0.79 (0.19 - 3.38)	0.755	0.25 (0.06 - 1.12)	0.07	2.78 (0.16 - 48.59)	0.484
College degree	1.65 (1.1 - 2.45)	0.014	0.93 (0.63 - 1.36)	0.705	2.37 (1.43 - 3.92)	0.001
Completed high school	1.27 (0.83 - 1.94)	0.27	0.88 (0.64 - 1.21)	0.427	1.85 (1.09 - 3.12)	0.022
No high school degree	0.6 (0.14 - 2.51)	0.48	1.03 (0.5 - 2.13)	0.941	0.48 (0.1 - 2.18)	0.338
Private insurance	1.13 (0.78 - 1.65)	0.521	0.72 (0.54 - 0.98)	0.034	2.2 (1.35 - 3.57)	0.001
Medicare	1.29 (0.88 - 1.91)	0.194	1.22 (0.84 - 1.76)	0.289	1.02 (0.62 - 1.66)	0.952

HR=hazard ratio, CI = confidence interval, FPL = federal poverty level. Cancer-specific survival can include cancer death from a cancer that was diagnosed after the time of survey completion. All analyses adjust for covariates including insurance status, marital status, sex, comorbidities, education, household income, geographic region, survey year (and, for analyses exclusively involving cancer survivors, additionally cancer site and time since cancer diagnosis).

© 2026 Barnes JM et al. *JAMA Network Open*.

eTable 4: Associations of high-deductible health plans with overall and cancer survival by cancer site and time since diagnosis subgroups

		OS		CSS	
		HR (95% CI)	P-value	HR (95% CI)	P-value
Cancer site	Breast	1.22 (0.77 - 1.92)	0.396	1.24 (0.67 - 2.32)	0.489
	Cervix	0.61 (0.19 - 1.97)	0.413	0.08 (0 - 1.67)	0.103
	Colon	0.86 (0.38 - 1.96)	0.719	0.98 (0.28 - 3.39)	0.972
	Lung	0.88 (0.39 - 1.97)	0.754	0.93 (0.35 - 2.45)	0.879
	Melanoma	1.08 (0.42 - 2.76)	0.873	0.85 (0.21 - 3.47)	0.818
	Prostate	1.83 (0.97 - 3.47)	0.063	2.12 (0.79 - 5.68)	0.135
	Other site	1.6 (1.12 - 2.29)	0.01	1.2 (0.76 - 1.9)	0.428
	Multiple cancers	1.58 (1.06 - 2.36)	0.024	1.98 (1.19 - 3.28)	0.009
Time since cancer diagnosis	>=16 years	1.08 (0.66 - 1.77)	0.77	0.75 (0.29 - 1.94)	0.55
	7-15 years	1.68 (1.16 - 2.44)	0.006	1.92 (1.17 - 3.17)	0.01
	3-6 years	1.49 (0.95 - 2.33)	0.082	1.54 (0.85 - 2.79)	0.158
	0-2 years	1.32 (0.91 - 1.9)	0.143	1.15 (0.74 - 1.79)	0.539

All analyses adjust for covariates including insurance status, marital status, sex, comorbidities, education, household income, geographic region, survey year, cancer site, and time since cancer diagnosis.

© 2026 Barnes JM et al. *JAMA Network Open*.

eTable 5: Characteristics of the study population with consideration of health savings account status (weighted %)

		HDHP without HSA		HDHP with HSA	
		Survivor (N=1,687)	No cancer history (N=24,541)	Survivor (N=644)	No cancer history (N=12,932)
Age	65-84	26.5	7.2	57.2	19.4
	40-64	64.7	54.8	37.7	44
	18-39	8.8	38	5.1	36.6
Race and ethnicity	Non-Hispanic White	83.3	74.4	82.7	68.9
	Non-Hispanic Black	6.4	8	7.7	10.8
	Non-Hispanic Other	5.1	7.3	4.3	8
	Hispanic	5.2	10.3	5.3	12.3
Insurance	Private	75.2	93.7	36.7	70.5
	Medicare	24.8	6.3	57.1	20.1
	Other/Not reported	0	0	6.2	9.4
Marital status	Not married	66.7	61.5	63.8	57.4
	Married	33.1	38.3	36.1	42.5
	Not reported	0.2	0.1	0.1	0.1
Sex	Male	38.5	49.6	43.1	49.3
	Female	61.5	50.4	56.9	50.7
Region	Northeast	14.2	14.3	19	18.6
	Midwest	26.3	25.1	23	22.4
	South	37.7	38.8	37	35.9
	West	21.7	21.8	21	23.1
Education	College degree	51.3	52.7	45.2	47.2
	Completed high school	43.8	41.6	44.7	43.9
	No high school degree	4.9	5.7	10.1	8.9
Poverty	>=400% FPL	50.8	49.9	42.8	43.6
	250-399% FPL	25.2	24	21.3	21.5
	125-249% FPL	14.3	15.3	18.3	17.5
	<125% FPL	3.4	4.4	7.3	9.2
	Unknown/not reported	6.3	6.3	10.2	8.2
Comorbidities	No	33.8	60.2	22.7	55.4
	Yes	66.2	39.8	77.3	44.6
Year	2011-2013	29.8	31.9	34.7	36.3
	2014-2016	39.4	39.2	38	38.3
	2017-2018	30.9	28.9	27.2	25.3
Cancer type	Breast	20.6	NA	20	NA
	Cervix	7.8		5.2	

© 2026 Barnes JM et al. *JAMA Network Open*.

	Colon	4		5.3	
	Lung	1.7		2.3	
	Melanoma	8.9		7.9	
	Multiple	13.7		16.2	
	Other/Not reported	34.1		29.3	
	Prostate	9.2		13.7	
Time since cancer diagnosis	>=16 years since diagnosis	19		21.9	
	7-15 years since diagnosis	27.8		29.1	
	3-6 years since diagnosis	24.8		24	
	0-2 years since diagnosis	27.5		23.5	
	Unknown/not reported	0.9		1.5	

© 2026 Barnes JM et al. *JAMA Network Open*.

eTable 6: Associations of high-deductible health plans and HSA status with overall survival, overall and by sociodemographic subgroups

	With HSA						Without HSA					
Subgroup	Cancer survivors		No history of cancer		Cancer survivors relative to those without cancer history		Cancer survivors		No history of cancer		Cancer survivors relative to those without cancer history	
	HR (95% CI)	P-value	HR (95% CI)	P-value	HR (95% CI)	P-value	HR (95% CI)	P-value	HR (95% CI)	P-value	HR (95% CI)	P-value
Overall	1.68 (1.03 - 2.74)	0.036	1 (0.78 - 1.28)	0.992	2.28 (1.33 - 3.9)	0.003	1.36 (1.1 - 1.69)	0.005	1.09 (0.97 - 1.23)	0.157	1.35 (1.05 - 1.73)	0.018
>=65 years	2.47 (0.91 - 6.69)	0.075	1.92 (1.02 - 3.62)	0.044	NA	NA	1.14 (0.88 - 1.48)	0.322	1.04 (0.88 - 1.23)	0.646	1.07 (0.79 - 1.46)	0.66
40-64 years	1.01 (0.59 - 1.73)	0.96	0.87 (0.65 - 1.17)	0.359	1.47 (0.79 - 2.72)	0.222	1.17 (0.81 - 1.7)	0.393	0.97 (0.82 - 1.15)	0.718	1.33 (0.91 - 1.97)	0.145
18-39 years	NA	NA	0.61 (0.27 - 1.38)	0.237	NA	NA	1.25 (0.2 - 7.9)	0.811	1.25 (0.74 - 2.12)	0.396	1.34 (0.27 - 6.73)	0.721
Male	1.75 (0.87 - 3.52)	0.117	1.25 (0.95 - 1.66)	0.116	1.73 (0.85 - 3.52)	0.133	1.28 (0.94 - 1.74)	0.114	1.15 (0.99 - 1.34)	0.07	1.2 (0.85 - 1.7)	0.293
Female	1.66 (0.89 - 3.11)	0.112	0.57 (0.35 - 0.94)	0.026	4.28 (2.02 - 9.06)	<.001	1.38 (1.02 - 1.87)	0.04	0.99 (0.82 - 1.2)	0.941	1.68 (1.17 - 2.42)	0.005
Non-Hispanic White	1.68 (1 - 2.84)	0.051	1.08 (0.82 - 1.41)	0.592	2.23 (1.25 - 3.97)	0.007	1.34 (1.06 - 1.71)	0.016	1.13 (0.99 - 1.28)	0.072	1.34 (1.02 - 1.76)	0.036
Non-Hispanic Black	5.41 (1.03 - 28.34)	0.046	0.76 (0.25 - 2.3)	0.632	2.74 (0.4 - 18.72)	0.304	1.08 (0.48 - 2.44)	0.848	0.81 (0.55 - 1.19)	0.284	0.96 (0.28 - 3.28)	0.948
Non-Hispanic Other	NA	NA	0.49 (0.1 - 2.37)	0.372	NA	NA	0.71 (0.05 - 9.84)	0.8	1.48 (0.77 - 2.84)	0.236	0.7 (0.23 - 2.16)	0.536
Hispanic	NA	NA	1.04 (0.4 - 2.68)	0.939	NA	NA	0.7 (0.23 - 2.1)	0.522	0.91 (0.57 - 1.43)	0.672	2.07 (0.57 - 7.47)	0.268
No comorbidities	1.14 (0.53 - 2.44)	0.741	0.92 (0.63 - 1.36)	0.686	1.75 (0.76 - 4.02)	0.191	1.6 (0.97 - 2.64)	0.065	0.87 (0.68 - 1.12)	0.282	2.11 (1.21 - 3.69)	0.008
Comorbidities	1.96 (1.09 - 3.54)	0.025	1.03 (0.75 - 1.43)	0.838	2.35 (1.2 - 4.61)	0.013	1.28 (1 - 1.63)	0.046	1.18 (1.03 - 1.35)	0.02	1.14 (0.87 - 1.49)	0.335
2011-2013 survey	1.58 (0.69 - 3.63)	0.277	1.07 (0.75 - 1.51)	0.713	1.92 (0.79 - 4.65)	0.147	1.37 (1.01 - 1.85)	0.043	1.13 (0.96 - 1.33)	0.135	1.31 (0.95 - 1.81)	0.097
2014-2016 survey	1.49 (0.74 - 3.02)	0.266	1.1 (0.74 - 1.63)	0.645	2.02 (0.91 - 4.47)	0.085	1.42 (1 - 2.03)	0.052	1.03 (0.82 - 1.29)	0.815	1.46 (0.97 - 2.2)	0.072

© 2026 Barnes JM et al. *JAMA Network Open*.

2017-2018 survey	2.06 (0.74 - 5.74)	0.168	0.56 (0.23 - 1.36)	0.199	5.83 (1.7 - 19.98)	0.005	1.17 (0.58 - 2.39)	0.659	1.07 (0.71 - 1.61)	0.749	1.05 (0.43 - 2.59)	0.915
>=400% FPL	1.51 (0.74 - 3.07)	0.254	1.19 (0.87 - 1.63)	0.272	1.93 (0.92 - 4.02)	0.08	1.55 (1.06 - 2.24)	0.022	1.02 (0.85 - 1.22)	0.872	1.8 (1.2 - 2.71)	0.005
250-399% FPL	2.63 (1.12 - 6.19)	0.027	0.82 (0.48 - 1.4)	0.475	3.4 (1.3 - 8.89)	0.012	1.23 (0.8 - 1.9)	0.345	1.04 (0.8 - 1.34)	0.78	1.17 (0.7 - 1.96)	0.548
125-249% FPL	NA	NA	0.57 (0.21 - 1.55)	0.269	NA	NA	1.31 (0.89 - 1.94)	0.17	1.2 (0.94 - 1.53)	0.144	1.02 (0.63 - 1.65)	0.936
<125% FPL	NA	NA	1.56 (0.47 - 5.16)	0.465	NA	NA	0.77 (0.31 - 1.89)	0.564	1.03 (0.62 - 1.72)	0.901	0.55 (0.11 - 2.68)	0.458
College degree	1.65 (0.89 - 3.05)	0.112	1.07 (0.75 - 1.52)	0.7	2.21 (1.12 - 4.39)	0.023	1.34 (0.96 - 1.86)	0.082	1.16 (0.95 - 1.4)	0.144	1.3 (0.89 - 1.9)	0.17
Completed high school	1.63 (0.72 - 3.69)	0.243	1.05 (0.74 - 1.51)	0.77	2.06 (0.88 - 4.85)	0.097	1.51 (1.12 - 2.05)	0.007	1.07 (0.9 - 1.28)	0.422	1.48 (1.04 - 2.11)	0.029
No high school degree	6.85 (0.8 - 58.37)	0.078	0.91 (0.21 - 3.95)	0.903	5.73 (0.52 - 62.98)	0.153	0.93 (0.45 - 1.93)	0.841	1.01 (0.74 - 1.38)	0.963	0.81 (0.36 - 1.84)	0.615
Private insurance	1.08 (0.63 - 1.86)	0.771	0.92 (0.71 - 1.2)	0.557	1.6 (0.86 - 3)	0.137	1.44 (1.02 - 2.03)	0.036	0.97 (0.83 - 1.14)	0.721	1.78 (1.24 - 2.55)	0.002
Medicare	3.61 (1.44 - 9.06)	0.006	1.53 (0.73 - 3.2)	0.256	2.63 (0.85 - 8.16)	0.094	1.05 (0.81 - 1.37)	0.701	1.21 (1.01 - 1.44)	0.036	0.85 (0.63 - 1.16)	0.305

NA = not applicable due to extremely limited number of events in one of the comparison groups leading to unstable estimates. All analyses adjust for covariates including insurance status, marital status, sex, comorbidities, education, household income, geographic region, survey year (and, for analyses exclusively involving cancer survivors, additionally cancer site and time since cancer diagnosis).

© 2026 Barnes JM et al. *JAMA Network Open*.

eTable 7: Associations of high-deductible health plans and HSA status with cancer-specific survival, overall and by sociodemographic subgroups

	With HSA						Without HSA					
Subgroup	Cancer survivors		No history of cancer		Cancer survivors relative to those without cancer history		Cancer survivors		No history of cancer		Cancer survivors relative to those without cancer history	
	HR (95% CI)	P-value	HR (95% CI)	P-value	HR (95% CI)	P-value	HR (95% CI)	P-value	HR (95% CI)	P-value	HR (95% CI)	P-value
Overall	1.42 (0.84 - 2.41)	0.193	1.09 (0.68 - 1.74)	0.728	2.29 (1.15 - 4.56)	0.018	1.27 (0.93 - 1.72)	0.13	0.85 (0.66 - 1.11)	0.233	1.79 (1.23 - 2.62)	0.002
>=65 years	2.93 (0.88 - 9.72)	0.079	2.3 (0.77 - 6.89)	0.137	NA	NA	1.16 (0.77 - 1.75)	0.477	0.92 (0.63 - 1.33)	0.652	1.2 (0.72 - 1.99)	0.487
40-64 years	0.81 (0.45 - 1.46)	0.484	0.88 (0.52 - 1.47)	0.619	1.23 (0.57 - 2.69)	0.596	1.09 (0.68 - 1.74)	0.735	0.68 (0.49 - 0.95)	0.024	1.8 (1.05 - 3.08)	0.033
18-39 years	NA	NA	NA	NA	0.06 (0.02 - 0.2)	<.001	1.1 (0.06 - 19.18)	0.946	1.35 (0.3 - 6.12)	0.698	0.97 (0.05 - 17.48)	0.984
Male	1.49 (0.73 - 3.05)	0.273	1.26 (0.66 - 2.39)	0.489	2.22 (0.86 - 5.71)	0.099	1.18 (0.75 - 1.87)	0.47	0.81 (0.56 - 1.18)	0.276	1.76 (1.03 - 3)	0.038
Female	1.33 (0.62 - 2.87)	0.46	0.92 (0.46 - 1.84)	0.818	2.39 (0.9 - 6.36)	0.082	1.35 (0.88 - 2.06)	0.173	0.89 (0.64 - 1.25)	0.502	1.86 (1.08 - 3.18)	0.024
Non-Hispanic White	1.39 (0.8 - 2.42)	0.244	1.05 (0.63 - 1.77)	0.847	2.5 (1.2 - 5.2)	0.015	1.27 (0.91 - 1.77)	0.16	0.86 (0.64 - 1.14)	0.294	1.85 (1.22 - 2.81)	0.004
Non-Hispanic Black	3.86 (0.41 - 36.12)	0.237	1.24 (0.23 - 6.81)	0.802	NA	NA	1.1 (0.38 - 3.22)	0.863	0.58 (0.24 - 1.37)	0.212	1.89 (0.36 - 9.82)	0.45
Non-Hispanic Other	NA	NA	2.67 (0.47 - 15.3)	0.27	NA	NA	0.64 (0.03 - 15.09)	0.78	1.4 (0.48 - 4.08)	0.542	1.16 (0.19 - 7.15)	0.875
Hispanic	NA	NA	0.46 (0.06 - 3.44)	0.45	NA	NA	0.46 (0.06 - 3.66)	0.466	0.74 (0.27 - 2.04)	0.564	1.98 (0.3 - 13.3)	0.481
No comorbidities	1.49 (0.67 - 3.3)	0.324	1.08 (0.51 - 2.27)	0.848	2.13 (0.76 - 6.03)	0.153	1.57 (0.85 - 2.9)	0.152	0.61 (0.35 - 1.05)	0.072	2.8 (1.26 - 6.24)	0.012

© 2026 Barnes JM et al. *JAMA Network Open*.

Comorbidities	1.35 (0.67 - 2.72)	0.401	1.07 (0.59 - 1.93)	0.827	2.1 (0.87 - 5.08)	0.101	1.19 (0.83 - 1.69)	0.346	0.97 (0.72 - 1.3)	0.816	1.47 (0.97 - 2.23)	0.066
2011-2013 survey	0.61 (0.17 - 2.24)	0.455	0.91 (0.49 - 1.7)	0.769	0.95 (0.23 - 3.89)	0.947	1.12 (0.7 - 1.8)	0.625	0.93 (0.67 - 1.28)	0.639	1.49 (0.91 - 2.44)	0.112
2014-2016 survey	1.71 (0.83 - 3.55)	0.146	1.61 (0.74 - 3.49)	0.227	1.4 (0.43 - 4.58)	0.576	1.48 (0.92 - 2.4)	0.109	0.76 (0.47 - 1.24)	0.277	2.18 (1.11 - 4.3)	0.024
2017-2018 survey	2.28 (0.75 - 6.93)	0.147	0.68 (0.08 - 5.58)	0.716	6.59 (0.74 - 58.92)	0.092	1.14 (0.5 - 2.6)	0.759	0.73 (0.31 - 1.72)	0.475	1.84 (0.53 - 6.34)	0.337
>=400% FPL	1.38 (0.69 - 2.75)	0.356	1.04 (0.55 - 1.95)	0.905	2.76 (1.12 - 6.81)	0.028	1.6 (1 - 2.57)	0.051	0.86 (0.58 - 1.29)	0.475	2.33 (1.32 - 4.11)	0.004
250-399% FPL	2.16 (0.79 - 5.92)	0.136	1.42 (0.61 - 3.31)	0.422	2.13 (0.61 - 7.51)	0.239	1.17 (0.65 - 2.13)	0.601	0.84 (0.51 - 1.39)	0.504	1.59 (0.75 - 3.38)	0.225
125-249% FPL	NA	NA	0.22 (0.03 - 1.62)	0.138	NA	NA	0.86 (0.43 - 1.69)	0.656	0.85 (0.47 - 1.51)	0.571	1.29 (0.55 - 2.99)	0.558
<125% FPL	NA	NA	NA	NA	0.21 (0.04 - 1)	0.05	0.8 (0.19 - 3.45)	0.769	0.28 (0.06 - 1.23)	0.092	2.4 (0.13 - 43.21)	0.552
College degree	2.13 (1.11 - 4.07)	0.022	1.21 (0.63 - 2.33)	0.565	3.44 (1.43 - 8.24)	0.006	1.34 (0.87 - 2.07)	0.186	0.85 (0.56 - 1.29)	0.451	1.9 (1.08 - 3.36)	0.027
Completed high school	0.53 (0.16 - 1.77)	0.301	1.29 (0.65 - 2.55)	0.463	0.74 (0.19 - 2.9)	0.663	1.44 (0.93 - 2.22)	0.099	0.81 (0.58 - 1.13)	0.218	2.13 (1.24 - 3.65)	0.006
No high school degree	30.95 (4.63 - 206.77)	<.001	NA	NA	NA	NA	0.29 (0.05 - 1.63)	0.159	1.18 (0.57 - 2.45)	0.659	0.22 (0.04 - 1.31)	0.096
Private insurance	0.93 (0.52 - 1.68)	0.814	0.91 (0.54 - 1.52)	0.715	1.6 (0.73 - 3.48)	0.237	1.2 (0.78 - 1.86)	0.414	0.7 (0.51 - 0.95)	0.023	2.17 (1.3 - 3.62)	0.003
Medicare	3.16 (0.98 - 10.21)	0.055	3.07 (1.07 - 8.81)	0.038	1.33 (0.29 - 6.1)	0.715	1.16 (0.77 - 1.75)	0.481	1.11 (0.75 - 1.64)	0.598	0.99 (0.59 - 1.66)	0.974

NA = not applicable due to extremely limited number of events in one of the comparison groups leading to unstable estimates. All analyses adjust for covariates including insurance status, marital status, sex, comorbidities, education, household income, geographic region, survey year (and, for analyses exclusively involving cancer survivors, additionally cancer site and time since cancer diagnosis).

© 2026 Barnes JM et al. *JAMA Network Open*.

eTable 8: Associations of high-deductible health plans and HSA status with survival by cancer site and time since diagnosis subgroups.

	With HSA				Without HSA			
Subgroup	OS		CSS		OS		CSS	
	HR (95% CI)	P-value	HR (95% CI)	P-value	HR (95% CI)	P-value	HR (95% CI)	P-value
Breast	2.5 (0.9 - 6.89)	0.078	2.05 (0.6 - 6.95)	0.249	1.04 (0.63 - 1.7)	0.881	1.05 (0.54 - 2.07)	0.879
Cervix	NA	NA	NA	NA	0.86 (0.28 - 2.67)	0.796	0.05 (0.01 - 0.53)	0.013
Colon	1.02 (0.12 - 8.82)	0.984	1.18 (0.13 - 11.1)	0.883	0.87 (0.36 - 2.1)	0.755	1.05 (0.27 - 4.14)	0.947
Lung	NA	NA	NA	NA	0.97 (0.45 - 2.07)	0.928	1 (0.38 - 2.61)	0.996
Melanoma	1.08 (0.19 - 6.31)	0.928	2.21 (0.28 - 17.39)	0.453	1.07 (0.37 - 3.07)	0.9	0.71 (0.2 - 2.5)	0.597
Prostate	3.02 (0.69 - 13.11)	0.141	2.31 (0.47 - 11.4)	0.304	1.64 (0.86 - 3.12)	0.136	2.03 (0.74 - 5.57)	0.17
Other site	1.37 (0.6 - 3.13)	0.462	0.8 (0.31 - 2.07)	0.641	1.53 (1.06 - 2.23)	0.025	1.3 (0.78 - 2.18)	0.31
Multiple cancers	2.01 (0.76 - 5.29)	0.16	2.17 (0.79 - 5.96)	0.133	1.42 (0.93 - 2.18)	0.106	1.67 (0.95 - 2.96)	0.076
>=16 years since diagnosis	0.56 (0.07 - 4.26)	0.576	NA	NA	1.15 (0.7 - 1.88)	0.594	0.9 (0.36 - 2.28)	0.824
7-15 years since diagnosis	1.71 (0.52 - 5.67)	0.379	1.08 (0.25 - 4.68)	0.916	1.59 (1.12 - 2.25)	0.009	1.98 (1.21 - 3.26)	0.007
3-6 years since diagnosis	2.17 (0.82 - 5.74)	0.117	1.9 (0.59 - 6.1)	0.281	1.32 (0.8 - 2.18)	0.272	1.34 (0.67 - 2.69)	0.41
0-2 years since diagnosis	1.31 (0.69 - 2.47)	0.407	1.36 (0.7 - 2.65)	0.36	1.24 (0.84 - 1.85)	0.282	1.05 (0.64 - 1.72)	0.852

NA = not applicable due to extremely limited number of events in one of the comparison groups leading to unstable estimates. All analyses adjust for covariates including insurance status, marital status, sex, comorbidities, education, household income, geographic region, survey year, cancer site, and time since cancer diagnosis.

© 2026 Barnes JM et al. *JAMA Network Open*.

eTable 9: Mediation analyses evaluating the role of barriers to care on the associations between HDHP with or without HSA and survival

Survival	Barrier to care	Association between HDHP and Barrier to Care	Association between barrier to care and survival	Association between HDHP and survival, adjusting for barrier to care		Mediation effect		
		OR (95% CI)	HR (95% CI)	HR (95% CI)	P-value	HR (95% CI)	P-value	% Mediated
		With HSA						
OS	General financial worry	0.91 (0.68, 1.23)	1.75 (1.44, 2.14)	1.62 (0.94, 2.8)	0.081	0.95 (0.8, 1.12)	0.546	-11.9
	Delayed or forgone care	1.16 (0.8, 1.67)	1.84 (1.48, 2.28)	1.67 (1.03, 2.7)	0.038	1.09 (0.87, 1.37)	0.447	14.6
	Cost-related medication underuse	1 (0.7, 1.44)	1.77 (1.47, 2.15)	1.58 (0.95, 2.64)	0.079	1 (0.81, 1.23)	0.996	-0.1
	Worry about medical bills	1.46 (1.04, 2.04)	1.71 (1.4, 2.08)	1.69 (1.04, 2.74)	0.034	1.22 (1.01, 1.48)	0.044	27.6
CSS	General financial worry	0.91 (0.68, 1.23)	1.66 (1.27, 2.17)	1.77 (0.98, 3.19)	0.059	0.95 (0.82, 1.11)	0.549	-8.9
	Delayed or forgone care	1.16 (0.8, 1.67)	1.99 (1.48, 2.67)	1.41 (0.83, 2.39)	0.199	1.1 (0.85, 1.43)	0.449	22.3
	Cost-related medication underuse	1 (0.7, 1.44)	1.86 (1.44, 2.41)	1.25 (0.71, 2.21)	0.435	1 (0.8, 1.25)	0.996	-0.2
	Worry about medical bills	1.46 (1.04, 2.04)	1.97 (1.48, 2.62)	1.44 (0.85, 2.43)	0.175	1.29 (1, 1.66)	0.049	41.2
		Without HSA						

© 2026 Barnes JM et al. *JAMA Network Open*.

OS	General financial worry	1.8 (1.51, 2.15)	1.67 (1.27, 2.18)	1.4 (1.06, 1.84)	0.017	1.35 (1.13, 1.62)	0.001	47.1
	Delayed or forgone care	2.17 (1.74, 2.7)	2.17 (1.56, 3.02)	1.31 (1.05, 1.63)	0.017	1.82 (1.34, 2.48)	<.001	69
	Cost-related medication underuse	2.1 (1.68, 2.62)	1.97 (1.52, 2.55)	1.34 (1.08, 1.67)	0.008	1.65 (1.29, 2.11)	<.001	62.9
	Worry about medical bills	1.66 (1.32, 2.08)	1.96 (1.5, 2.56)	1.35 (1.08, 1.67)	0.007	1.4 (1.15, 1.72)	0.001	53.3
CSS	General financial worry	1.8 (1.51, 2.15)	1.54 (1.08, 2.21)	1.37 (0.94, 1.98)	0.098	1.29 (1.03, 1.61)	0.026	45
	Delayed or forgone care	2.17 (1.74, 2.7)	2.26 (1.5, 3.4)	1.22 (0.89, 1.66)	0.215	1.88 (1.3, 2.7)	0.001	76.2
	Cost-related medication underuse	2.1 (1.68, 2.62)	2.01 (1.46, 2.77)	1.27 (0.93, 1.73)	0.131	1.67 (1.26, 2.23)	<.001	68.3
	Worry about medical bills	1.66 (1.32, 2.08)	2.32 (1.62, 3.32)	1.24 (0.92, 1.69)	0.163	1.53 (1.18, 1.99)	0.002	66.2

All analyses adjust for covariates including insurance status, marital status, sex, comorbidities, education, household income, geographic region, survey year (and, for analyses exclusively involving cancer survivors, additionally cancer site and time since cancer diagnosis).

© 2026 Barnes JM et al. *JAMA Network Open*.

eFigure 1: Assumed causal pathway for mediation analyses

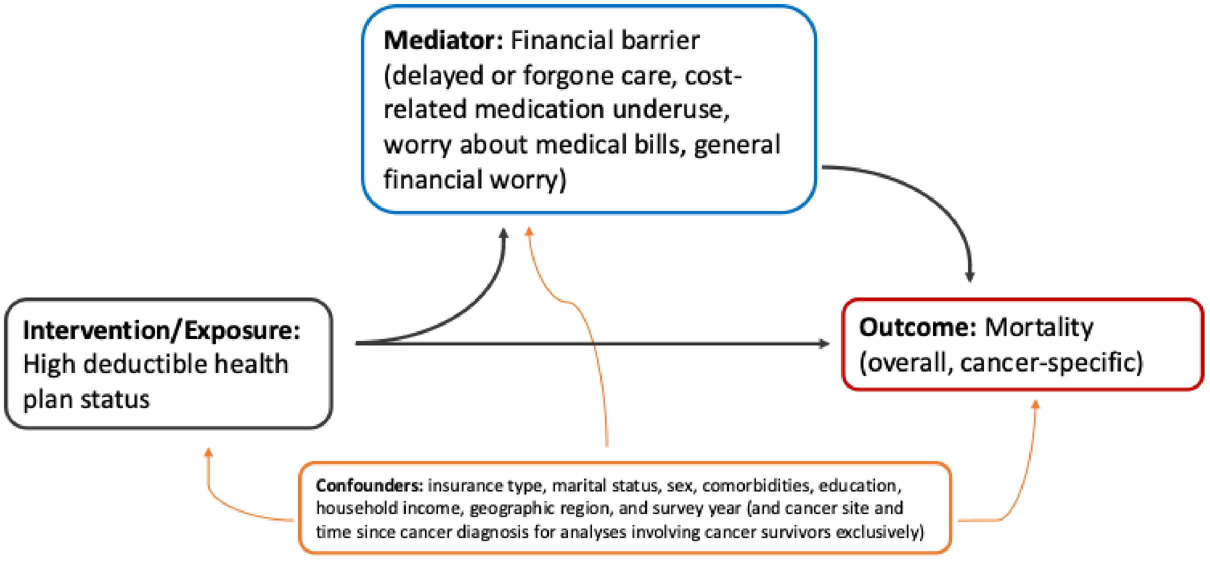

© 2026 Barnes JM et al. *JAMA Network Open*.

eFigure 2: Derivation of the dataset for analyses

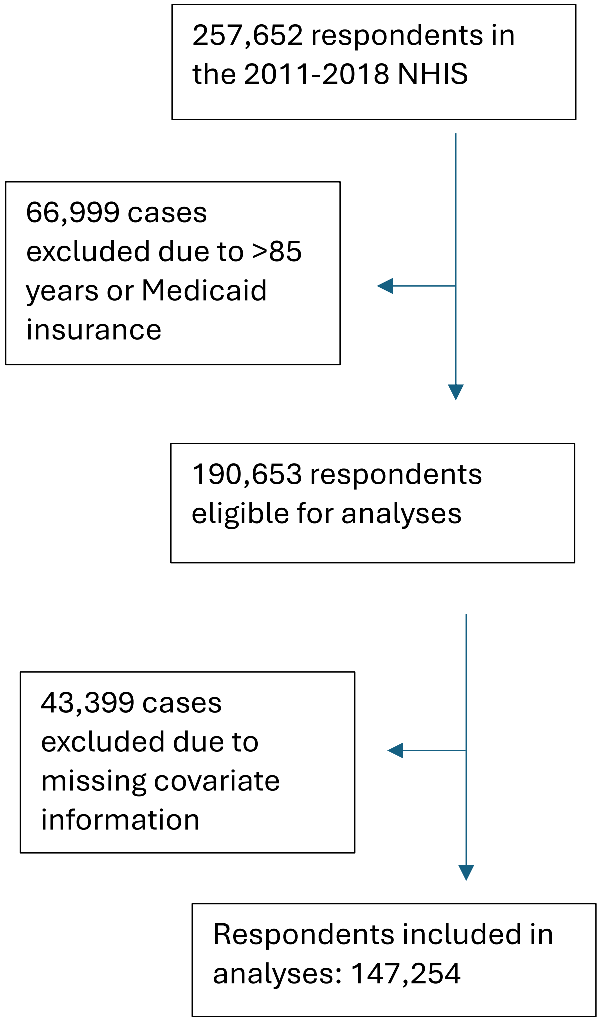

© 2026 Barnes JM et al. *JAMA Network Open*.
