# Supplementary material for: High-Deductible Health Plans and Mortality Among Cancer Survivors
Source: JAMA Netw Open. 2026 Jan 29;9(1):e2556451. doi: 10.1001/jamanetworkopen.2025.56451 (PMC12856683; doi:10.1001/jamanetworkopen.2025.56451)
Supplement: Supplement 2. — Data Sharing Statement [file jamanetwopen-e2556451-s002.pdf]

Data Sharing Statement

Barnes. High-Deductible Health Plans and Mortality Among Cancer Survivors. *JAMA Netw Open*. Published January 29, 2026. doi:10.1001/jamanetworkopen.2025.56451

Data
Data available: No

Additional Information
Explanation for why data not available: The data are already publicly available.
